# Depemokimab demonstrates efficacy in patients with type 2 asthma with comorbid CRSwNP: Phase III SWIFT-1/-2 analysis

**DOI:** 10.3389/falgy.2026.1766647

**Published:** 2026-03-06

**Authors:** Enrico Heffler, Diana Jarreta, Chang-Qing Zhu, Anna Vichiendilokkul, Peter Howarth, Anju Peters, Santiago Quirce, David J. Jackson

**Affiliations:** 1 Department of Biomedical Sciences, Humanitas University, Pieve Emanuele, Italy; 2 Personalized Medicine, Asthma and Allergy Unit, IRCCS Humanitas Research Hospital, Rozzano, Italy; 3 Clinical Science, GSK, Madrid, Spain; 4 Biostatistics, GSK, London, United Kingdom; 5 Global Medical Affairs, GSK, Collegeville, PA, United States; 6 Global Medical Affairs, Respiratory Specialty Care, GSK, London, United Kingdom; 7 Division of Allergy and Immunology, Department of Medicine, Northwestern University Feinberg School of Medicine, Chicago, IL, United States; 8 Department of Allergy, La Paz University Hospital, IdiPAZ, Madrid, Spain; 9 Department Guy’s Severe Asthma Centre, School of Immunology & Microbial Sciences, King’s College London, Guy’s Hospital, King’s College London, London, United Kingdom

**Keywords:** ACQ-5, anti-IL-5, asthma, biologics, chronic rhinosinusitis with nasal polyps, depemokimab, SGRQ, type 2 inflammation

## Abstract

**Introduction:**

The efficacy of twice-yearly depemokimab was demonstrated in the Phase III SWIFT-1/-2 trials for type 2 asthma characterized by blood eosinophils, and ANCHOR-1/-2 trials for chronic rhinosinusitis with nasal polyps (CRSwNP). Up to 40% of patients with severe asthma are estimated to have comorbid CRSwNP, an overlap associated with increased disease burden. Depemokimab may therefore offer meaningful clinical benefit in this subpopulation. This analysis evaluated the efficacy of depemokimab in patients with type 2 asthma and comorbid CRSwNP using pooled data from SWIFT-1/-2.

**Methods:**

Patients with type 2 asthma were randomized 2:1 to receive depemokimab 100 mg subcutaneously or placebo, plus standard of care, every 26 weeks for 52 weeks. Pre-specified outcomes included annualized exacerbation rates over 52 weeks, and St George's Respiratory Questionnaire (SGRQ) and Asthma Control Questionnaire-5 (ACQ-5) scores over 52 weeks, analyzed by baseline comorbid CRSwNP subgroup.

**Results:**

In the asthma with CRSwNP subgroup, the annualized rate of exacerbations over 52 weeks was lower in patients who received depemokimab [0.51 (95% CI: 0.34, 0.75); *n* = 80] vs. placebo [1.61 (95% CI: 0.99, 2.60); *n* = 33], with a rate ratio (95% CI) of 0.31 (0.17, 0.58). Additionally, an improvement in least squares (LS) mean SGRQ total score in the depemokimab group vs. placebo was noted after 4 weeks of treatment [−7.12-point difference (95% CI: −13.41, −0.83)] which was sustained up to 52 weeks [−8.32-point difference (95% CI: −15.77, −0.88)] in the asthma with CRSwNP subgroup. Similarly, an improvement from baseline in LS mean ACQ-5 score was noted with depemokimab vs. placebo after 2 weeks of treatment [−0.40-point difference (95% CI: −0.79, −0.01)], which was maintained up to 52 weeks [−0.76-point difference at (95% CI: −1.23, −0.28)] in the asthma with CRSwNP subgroup.

**Discussion:**

Twice-yearly depemokimab reduced annualized exacerbation rates and demonstrated greater improvements in SGRQ and ACQ-5 scores vs. placebo in patients with type 2 asthma and comorbid CRSwNP. These improvements were greater than those reported in the overall SWIFT-1/-2 population. These findings identify that type 2 asthma with CRSwNP is a clinically recognizable phenotype that is likely to have enhanced benefit with depemokimab therapy.

**Clinical trial identifiers:**

NCT04719832/NCT04718103.

## Graphical Abstract

**Figure:**
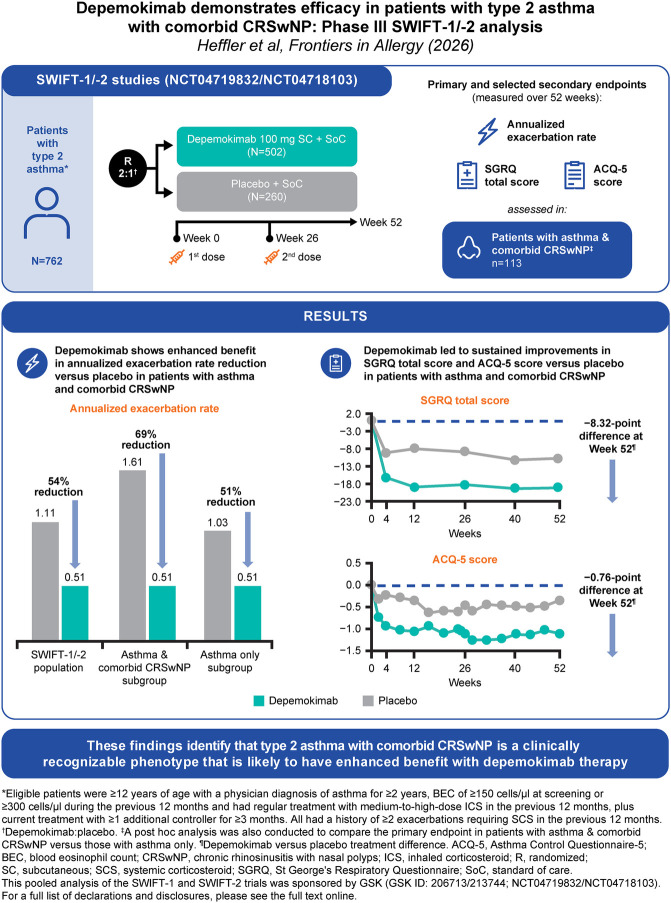
Graphical abstract.

## 1 Introduction

Asthma and chronic rhinosinusitis with nasal polyps (CRSwNP) are inflammatory airway diseases primarily driven by type 2 inflammation in the majority of cases (1, 2). Type 2 inflammation is associated with reduced lung function and increased risk of exacerbations in patients with asthma and correlates with a greater disease severity in those with CRSwNP (2–4). Due to shared underlying disease mechanisms, CRSwNP is a common comorbidity in patients with type 2 asthma (4), adding to both the patient and economic burden of the disease (2, 5, 6). Recent real-world data from the Severe Asthma Network in Italy (SANI) registry showed that approximately 38.1%–40.6% of patients with severe asthma also have comorbid CRSwNP; this subgroup of patients experience an increased risk of exacerbations and oral corticosteroid (OCS) use, resulting in reduced patient quality of life and increased healthcare costs (5–7). As such, effective treatments for both these conditions represent a key medical need.

Type 2 inflammation is primarily mediated by cytokines such as interleukin (IL)-5, IL-4 and IL-13 (1). In particular, IL-5 promotes the maturation, activation, proliferation, migration and survival of eosinophils, leading to its identification as a key factor in the pathophysiology and progression of inflammatory airway diseases such as type 2 asthma, characterized by increased blood and airway eosinophils, and CRSwNP (1, 3). More recently, it has been recognized that the role of IL-5 extends beyond its association with eosinophils, as IL-5 inhibition has been shown to affect a wide range of cell types involved in immune and structural airway function, including basophils, neutrophils, mast cells, B cells/plasma cells, epithelial cells, airway smooth muscle cells, and bronchial fibroblasts (8). As such, IL-5 is thought to have a broader role in airway inflammation, airway remodeling and airway smooth muscle thickening (8). In recent decades, several biologic therapies targeting IL-5 and other type 2 inflammatory mediators have been developed and approved as add-on treatments for moderate-to-severe (omalizumab, dupilumab) or severe (mepolizumab, tezepelumab) asthma and CRSwNP (1, 9–12).

Depemokimab is the first ultra-long-acting biologic engineered to have enhanced IL-5 binding affinity, high potency, and an extended half-life (13–15) enabling twice-yearly dosing and sustained suppression of type 2 inflammation (15). In the Phase III SWIFT-1/-2 trials, depemokimab 100 mg administered subcutaneously every 26 weeks reduced the annualized rate of exacerbations in patients with type 2 asthma characterized by blood eosinophils by 54% compared with placebo (16). In tandem, a rapid and sustained reduction in blood eosinophil counts was observed with depemokimab vs. placebo, with reductions from baseline of 83% in SWIFT-1 and 82% in SWIFT-2 at Week 52 (16). Depemokimab also demonstrated a favorable safety profile, with overall adverse event rates in SWIFT-1 and SWIFT-2 (73% and 72%, respectively), similar to those observed with placebo (73% and 78%, respectively) (16).

Targeting IL-5 in CRSwNP is thought to mitigate the impact of chronic type 2 inflammation by rebalancing immune responses, reducing nasal polyp formation and restoring epithelial integrity, ultimately reducing symptomatic burden, preventing disease recurrence and the need for surgery, and improving patient quality of life (3). In the Phase III ANCHOR-1/-2 trials in patients with CRSwNP, depemokimab led to a nominally significant reduction vs. placebo in total endoscopic nasal polyps score [−0.7 points; 95% confidence interval (CI): −0.9, −0.4] and nasal obstruction verbal response score (-0.24 points; 95% CI: −0.39, −0.08). Both of these coprimary endpoints reached statistical significance in the individual ANCHOR-1 and ANCHOR-2 trials. Nominally significant improvements were also observed in ANCHOR-1/-2 in the Sino-Nasal Outcome Test-22 score (SNOT-22: −8.1 points; 95% CI: −13.9, −2.3) and Asthma Control Questionnaire-5 score (ACQ-5: −0.75 points; 95% CI: −1.26, −0.25). The adverse event profile of depemokimab in ANCHOR-1/-2 was similar to that observed with placebo (75% vs. 79%, respectively) (17).

Given the high prevalence of comorbid CRSwNP among patients with type 2 asthma characterized by blood eosinophils, and its association with greater disease burden, this pooled analysis of SWIFT-1/-2 data was conducted with the aim of evaluating whether twice-yearly depemokimab provides clinically meaningful benefits in this subpopulation.

## 2 Methods

### 2.1 Study design

The SWIFT-1/-2 trials (GSK study numbers: 206713/213744; NCT04719832/NCT04718103) (16) were Phase III, multicenter, randomized, double-blind, placebo-controlled replicate trials, conducted from February 4, 2021, to April 11, 2024. The full study design of SWIFT-1/-2 trials has been published previously (16). Briefly, patients were randomized 2:1 to receive depemokimab 100 mg subcutaneously or placebo, in addition to standard of care, every 26 weeks for 52 weeks. The use of rescue medication (albuterol/salbutamol metered-dose inhalers) was permitted throughout the study. The objectives of SWIFT-1/-2 were to investigate the efficacy and safety of depemokimab in patients with type 2 asthma, characterized by blood eosinophil counts (16). However, patients with comorbid CRSwNP were also permitted to enroll (16).

Both trials were conducted in accordance with the International Conference on Harmonisation Good Clinical Practice guidelines, the principles of the Declaration of Helsinki, and all applicable national and international laws and regulations. All patients provided written informed consent.

### 2.2 Patients

Eligible patients enrolled in SWIFT-1/-2 were ≥12 years of age with a diagnosis of asthma (with or without past or current comorbid CRSwNP), a blood eosinophil count of at least 150 cells/μL at screening (or at least 300 cells/μL during the 12 months prior to the study), had airflow obstruction despite receiving regular treatment with medium-/high-dose inhaled corticosteroids (ICS), had received ≥1 additional controller for ≥3 months, and had a history of ≥2 exacerbations in the 12 months prior to the study (16). Both symptomatic and non-symptomatic patients were permitted for the inclusion of all baseline ACQ-5 scores. Patients receiving a biologic therapy as part of their current maintenance therapy were excluded, as were patients who had received anti-IL-5 antibody therapy within the past 12 months, or dupilumab or omalizumab within the past 130 days. Full eligibility criteria have been published previously (16).

### 2.3 Endpoints and assessments

The pre-specified primary endpoint was the annualized rate of clinically significant asthma exacerbations during the 52-week study period, defined as any worsening of asthma leading to the use of systemic corticosteroids (or a doubling or more of the dose for ≥3 days in patients who were already receiving OCS), hospitalization, or an emergency department visit (16). In addition to the overall SWIFT-1/-2 population analysis, a pre-specified analysis of the primary endpoint and selected secondary endpoints [change from baseline in St George's Respiratory Questionnaire (SGRQ) total score, ACQ-5 score, and pre-bronchodilator forced expiratory volume in 1 s (FEV_1_)] was carried out among patients with past or current comorbid CRSwNP (asthma with CRSwNP subgroup). A pre-specified analysis of the SNOT-22 score was carried out in patients who had current CRSwNP at baseline. Additionally, a *post hoc* analysis compared the primary endpoint in patients in the asthma with CRSwNP subgroup vs. patients without comorbid CRSwNP (asthma-only subgroup).

### 2.4 Statistical analysis

The primary efficacy endpoint (the annualized rate of clinically significant exacerbations) was analyzed using a generalized linear model assuming a negative binomial distribution. The model included covariates of treatment group, baseline ICS dose (medium or high), exacerbation history (2, 3, ≥4), geographical region, sex, baseline pre-bronchodilator percent predicted FEV_1_, study (SWIFT-1 or SWIFT-2), subgroup (patients with type 2 asthma with/without current or past comorbid CRSwNP at baseline), and subgroup by treatment group. Change from baseline in SGRQ total score, ACQ-5, pre-bronchodilator FEV_1_, and SNOT-22 were analyzed using a repeated measures model with covariates of treatment group, baseline ICS dose (medium or high), exacerbation history (2, 3, 4+), geographical region, baseline outcome score, baseline pre-bronchodilator percent predicted FEV_1_, study (SWIFT-1 or SWIFT-2), visit by baseline outcome score, and visit by treatment group. No analyses were adjusted for multiplicity.

SNOT-22 data were also analyzed by baseline severity using a cutoff value of 40 (patients with <40 and ≥40 in the asthma with CRSwNP subgroup), which is a recognized threshold for significantly impaired quality of life and one of the criterion for biologic initiation in CRSwNP (18). SNOT-22 score was analyzed using a repeated measures model with covariates of treatment group, baseline ICS dose (medium or high), exacerbation history (2, 3, 4+), geographical region, baseline pre-bronchodilator percent predicted FEV_1_, study (206713 or 213744), visit, visit by treatment group, baseline SNOT-22 score category (<40 and ≥40), baseline SNOT-22 score category by treatment, and baseline SNOT-22 score category by treatment by visit. An analysis of proportion of responders according to baseline SNOT-22 score was also performed. Response was defined as a reduction in SNOT-22 total score of at least 8.9 units compared with baseline. Non-response was defined as a SNOT-22 total score not meeting the 8.9-point reduction compared with baseline or a missing SNOT-22 total score with no subsequent on-treatment scores. Subjects did not have a responder status derived at a particular visit if the SNOT-22 score was missing but subsequent on-treatment SNOT-22 scores were present. The responder analysis was performed using a generalized linear mixed model with a logit link function and covariates of treatment group, baseline ICS dose (medium or high), exacerbation history (2, 3, 4+), geographical region, baseline pre-bronchodilator percent predicted FEV_1_, study (206713 or 213744), baseline SNOT-22 total score, visit, visit by baseline SNOT-22 total score, and visit by treatment group.

### 2.5 Data sharing statement

Please refer to GSK weblink to access GSK's data sharing policies and as applicable seek anonymized patient level data via the link https://www.gsk-studyregister.com/en/.

## 3 Results

### 3.1 Patient population

In total, 113/762 (14.8%) patients with type 2 asthma from the SWIFT-1/-2 trials were included in the asthma with CRSwNP subgroup. Of these, 80 (70.8%) were randomized to receive depemokimab 100 mg subcutaneously and 33 (29.2%) to receive placebo. Baseline patient characteristics for the asthma with CRSwNP subgroup, as well as the asthma-only subgroup and the overall SWIFT-1/-2 population, are presented in Table 1, and were broadly similar across treatment groups. Key differences between patients in the asthma with CRSwNP subgroup and the asthma-only subgroup included a higher geometric mean blood eosinophil count (431 cells/µL vs. 304 cells/µL, respectively) and a greater proportion of patients receiving high-dose ICS (63% vs. 55%, respectively). The proportion of patients using maintenance OCS at baseline was 5% in both subgroups.

**Table 1:** Baseline demographics and characteristics for patients with type 2 asthma in the overall pooled SWIFT-1/-2 population with and without past or current comorbid CRSwNP.

Baseline demographics and clinical characteristics	SWIFT-1/-2 population			CRSwNP subgroup			Asthma-only subgroup		
	Depemokimab 100 mg SC	Placebo	Overall	Depemokimab 100 mg SC	Placebo	Overall	Depemokimab 100 mg SC	Placebo	Overall
	(*N* = 502)	(*N* = 260)	(*N* = 762)	(*n* = 80)	(*n* = 33)	(*N* = 113)	(*n* = 422)	(*n* = 227)	(*N* = 649)
Baseline demographics									
Age, mean (SD), years	53.8 (14.9)	52.4 (15.8)	53.3 (15.2)	55.3 (11.5)	52.6 (13.3)	54.5 (12.1)	53.5 (15.5)	52.4 (16.1)	53.1 (15.7)
Female, *n* (%)	304 (61)	160 (62)	464 (61)	50 (63)	18 (55)	68 (60)	254 (60)	142 (63)	396 (61)
BMI, mean (SD), kg/m^2^	28.3 (5.9)	28.6 (6.6)	28.4 (6.1)	27.8 (5.1)	29.3 (7.1)	28.2 (5.8)	28.4 (6.0)	28.5 (6.5)	28.4 (6.2)
Asthma duration, mean (SD), years	24.0 (17.5)	22 (17.2)	23.4 (17.5)	23.2 (16.7)	25.5 (18.5)	23.9 (17.2)	24.2 (17.7)	21.5 (17.0)	23.3 (17.5)
Clinical characteristics									
ICS dose, *n* (%)									
Medium	212 (42)	121 (47)	333 (44)	33 (41)	9 (27)	42 (37)	179 (42)	112 (49)	291 (45)
High	290 (58)	139 (53)	429 (56)	47 (59)	24 (73)	71 (63)	115 (51)	243 (58)	358 (55)
Maintenance OCS, *n* (%)	21 (4)	19 (7)	40 (5)	4 (5)	2 (6)	6 (5)	17 (4)	17 (7)	34 (5)
Blood eosinophil count, Gmean (log SD), cells/µL	318 (0.84)	319 (0.83)	318 (0.84)	415 (0.79)	471 (0.78)	431 (0.78)	305 (0.82)	302 (0.82)	304 (0.82)
Number of exacerbations requiring OCS/SCS in previous 12 months, mean (SD)	2.4 (1.6)	2.4 (1.2)	2.4 (1.5)	2.5 (1.0)	2.6 (1.4)	2.5 (1.1)	2.4 (1.7)	2.4 (1.1)	2.4 (1.5)
SGRQ total score, *n*	490	254	744	79	33	112	411	221	632
Mean (SD)	44.7 (19.7)	43.8 (19.8)	44.4 (19.7)	44.9 (19.2)	46.6 (19.8)	45.4 (19.3)	44.7 (19.8)	43.4 (19.9)	44.3 (19.8)
ACQ-5 score, *n*	490	254	744	79	33	112	411	221	632
Mean (SD)	2.2 (1.1)	2.2 (1.1)	2.2 (1.1)	2.1 (1.1)	2.5 (1.1)	2.2 (1.1)	2.2 (1.1)	2.2 (1.1)	2.2 (1.1)
Pre-bronchodilator FEV_1_, *n*	490	252	742	78	32	110	412	220	632
Mean (SD), L*	1.9 (0.7)	1.8 (0.7)	1.8 (0.7)	1.7 (0.6)	1.8 (0.6)	1.8 (0.6)	1.9 (0.7)	1.8 (0.7)	1.9 (0.7)
SNOT-22 score, n/N^†^	–	–	–	47/49	23/23	70/72	–	–	–
Mean (SD)	–	–	–	40.8 (22.4)	42.7 (21.8)	41.4 (22.1)	–	–	–

*Screening values were consistent with baseline values (1.7–1.9); ^†^patients with current medical condition of CRSwNP at baseline.

ACQ-5, Asthma Control Questionnaire-5; BMI, body mass index; CRSwNP, chronic rhinosinusitis with nasal polyps; FEV_1_, forced expiratory volume in 1 s; Gmean, geometric mean; ICS, inhaled corticosteroid; OCS, oral corticosteroid; SC, subcutaneous; SCS, systemic corticosteroid; SD, standard deviation; SGRQ, St George's Respiratory Questionnaire; SNOT-22, Sino-Nasal Outcome Test-22; US, United States.

### 3.2 Annualized rate of clinically significant asthma exacerbations

**Figure 1:**
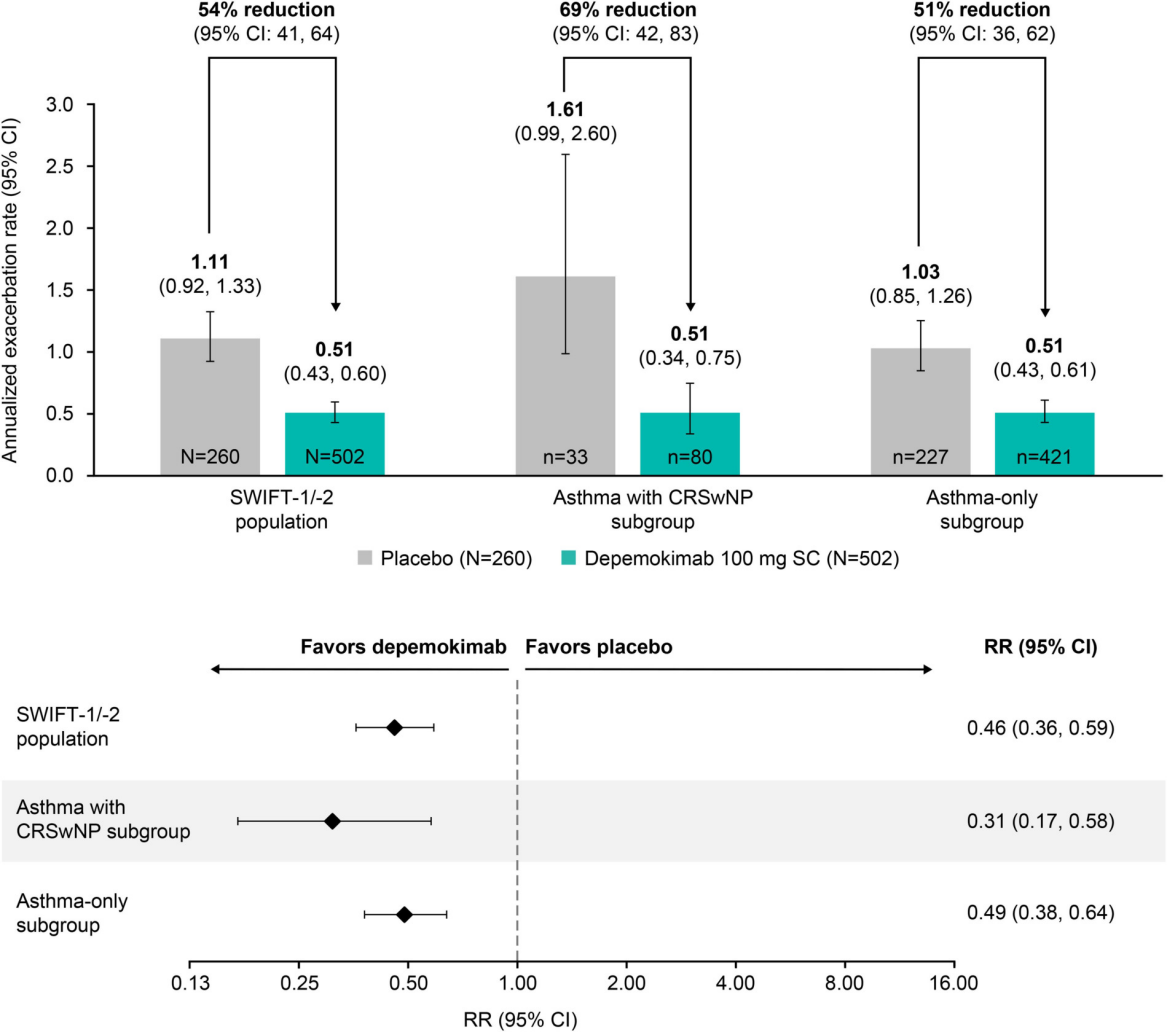
Annualized rate of clinically significant asthma exacerbations over 52 weeks by patients with type 2 asthma with and without past or current comorbid CRSwNP at baseline. Analysis performed using a generalized linear model assuming a negative binomial distribution and covariates of treatment group, baseline ICS dose (medium or high), exacerbation history (2, 3, 4+), geographical region, baseline pre-bronchodilator percent predicted FEV_1_, study (SWIFT-1 or SWIFT-2), past or current medical condition of CRSwNP (yes/ no) and past or current medical condition of CRSwNP by treatment group. CI, confidence interval; CRSwNP, chronic rhinosinusitis with nasal polyps; FEV_1_, forced expiratory volume in 1 s; ICS, inhaled corticosteroid; SC subcutaneous; RR, rate ratio.

The rate of clinically significant annualized exacerbations over 52 weeks was substantially lower in depemokimab-treated patients compared with placebo-treated patients in both the asthma with CRSwNP (0.51 vs. 1.61) and asthma-only (0.51 vs. 1.03) subgroups, as well as the overall SWIFT-1/-2 population (0.51 vs. 1.11) (Figure 1) (16). The treatment effect of depemokimab vs. placebo on the annualized exacerbation rate was more pronounced in the asthma with CRSwNP subgroup [rate ratio (95% CI): 0.31 (0.17, 0.58); 69% reduction] than in the asthma-only subgroup [rate ratio (95% CI): 0.49 (0.38, 0.64); 51% reduction] and overall SWIFT-1/-2 population [rate ratio (95% CI): 0.46 (0.36, 0.59); 54% reduction], albeit with overlapping CIs (Figure 1) (16).

### 3.3 SGRQ total score

**Figure 2:**
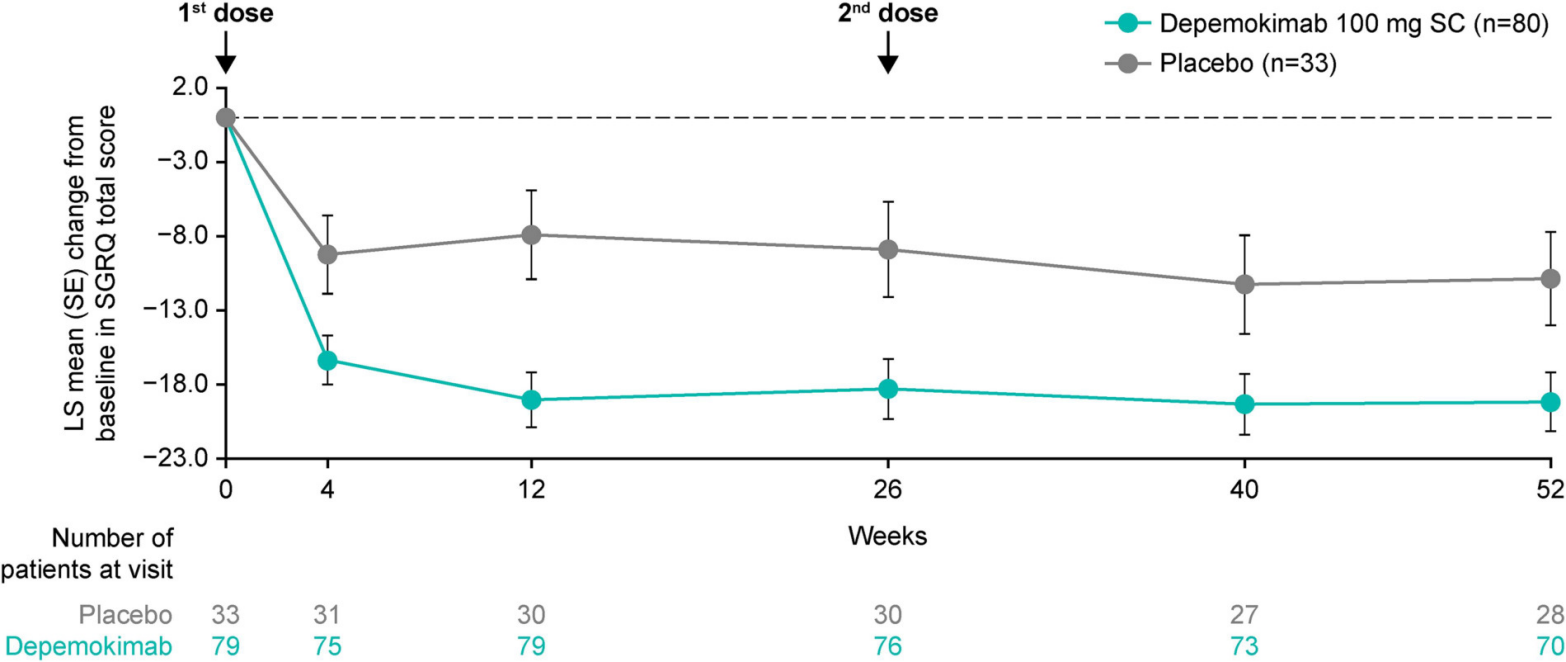
LS mean change from baseline in SGRQ total score over 52 weeks in patients with type 2 asthma with past or current comorbid CRSwNP at baseline. Analyses were performed using a repeated measures model with covariates of treatment group, baseline ICS dose (medium or high), exacerbation history (2, 3, 4+), geographical region, baseline SGRQ total score, baseline pre-bronchodilator percent predicted FEV_1_, visit by baseline SGRQ total score and visit by treatment group (depemokimab or placebo). For the pooled analysis, study (SWIFT-1 or SWIFT-2) was also included as an additional covariate. A greater reduction in SGRQ score indicates a greater improvement in patient outcomes. CRSwNP, chronic rhinosinusitis with nasal polyps; FEV_1_, forced expiratory volume in 1 s; ICS, inhaled corticosteroid; LS, least squares; SC, subcutaneous; SGRQ, St George's Respiratory Questionnaire; SE, standard error.

In the asthma with CRSwNP subgroup, depemokimab led to an improvement from baseline in LS mean SGRQ total score compared with placebo at the first assessment after 4 weeks of treatment [−7.12-point difference (95% CI: −13.41, −0.83)]. This improvement was sustained up to 52 weeks [−8.32-point difference at Week 52 (95% CI: −15.77, −0.88); Figure 2].

### 3.4 ACQ-5 score

**Figure 3:**
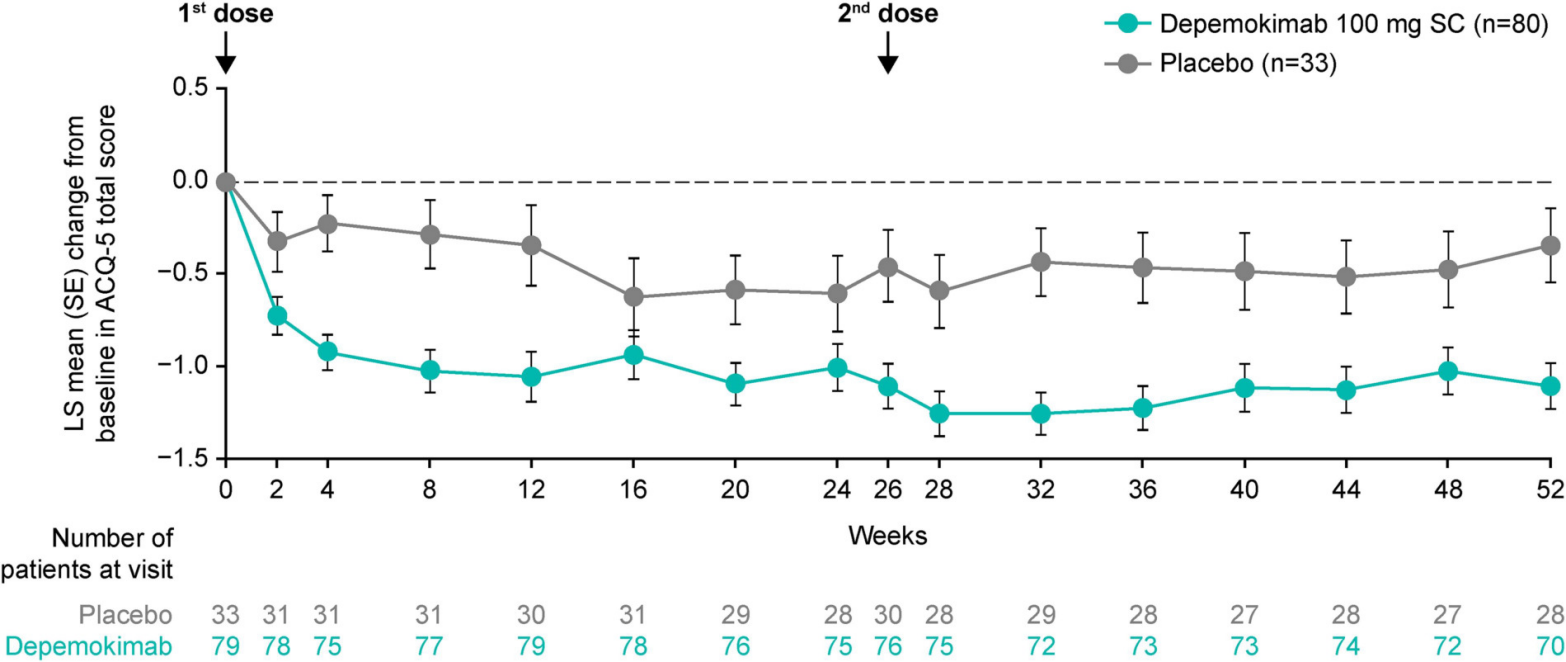
LS mean change from baseline in ACQ-5 score over 52 weeks in patients with type 2 asthma with past or current comorbid CRSwNP at baseline. Analyses were performed using a repeated measures model with covariates of treatment group, baseline ICS dose (medium or high), exacerbation history (2, 3, 4+), geographical region, baseline ACQ-5 score, baseline pre-bronchodilator percent predicted FEV_1_, visit by baseline ACQ-5 score and visit by treatment group (depemokimab or placebo). For the pooled analysis, study (SWIFT-1 or SWIFT-2) was also included as an additional covariate. A greater reduction in ACQ-5 score indicates a greater improvement in patient outcomes. ACQ-5, Asthma Control Questionnaire-5; CRSwNP, chronic rhinosinusitis with nasal polyps; FEV_1_, forced expiratory volume in 1 s; ICS, inhaled corticosteroid; LS, least squares; SC, subcutaneous; SE, standard error.

Similarly, in the asthma with CRSwNP subgroup, depemokimab led to an improvement from baseline in LS mean ACQ-5 score compared with placebo at the first assessment after 2 weeks of treatment [−0.40-point difference (95% CI: −0.79, −0.01)]. An improvement was maintained up to 52 weeks [−0.76-point difference at Week 52 (95% CI: −1.23, −0.28)], although some fluctuations were observed around Weeks 16–24 in both treatment groups (Figure 3). In patients receiving depemokimab, the LS mean change from baseline in ACQ-5 (19) exceeded the minimal clinically important difference (MCID; 0.5-point reduction) vs. placebo at most timepoints; depemokimab vs. placebo treatment difference (95% CI) was −0.66 (−1.12, −0.20) at Week 26 and −0.76 (−1.23, −0.28) at Week 52.

#### 3.4.1 Pre-bronchodilator FEV_1_

Improvements from baseline in LS mean (SE) pre-bronchodilator FEV_1_ were observed in the asthma with CRSwNP subgroup at Week 26 and Week 52 with both depemokimab [0.29 (0.057) and 0.31 (0.053), respectively] and placebo [0.23 (0.093) and 0.26 (0.088), respectively]. However, there was no substantial improvement in LS mean pre-bronchodilator FEV_1_ with depemokimab vs. placebo in the asthma with CRSwNP subgroup at either Week 26 or Week 52 [Week 26: 0.057 L difference (95% CI: −0.161, 0.276); Week 52: 0.057 L difference (95% CI: −0.148, 0.262); Figure E1 in the Online Repository].

### 3.5 SNOT-22 score

**Figure 4:**
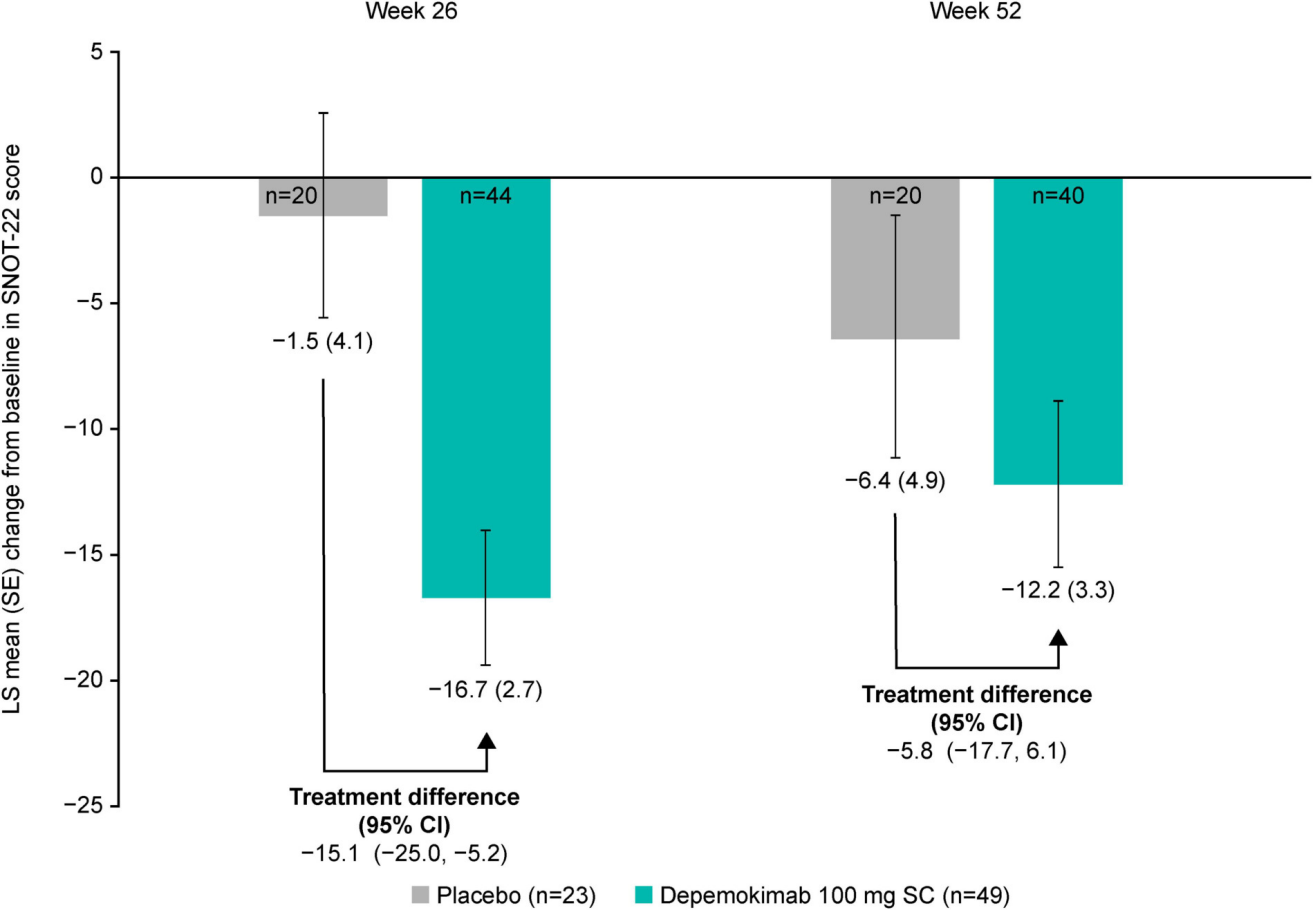
LS mean change from baseline in SNOT-22 score at weeks 26 and 52 in patients with type 2 asthma with current comorbid CRSwNP at baseline. *Only patients with current CRSwNP at baseline were included in the analysis. Analyses were performed using a repeated measures model with covariates of treatment group, baseline ICS dose (medium or high), exacerbation history (2, 3, 4+), geographical region, baseline SNOT-22 score, baseline pre-bronchodilator percent predicted FEV_1_, visit by baseline SNOT-22 total score and visit by treatment group (depemokimab or placebo). For the pooled analysis, study (SWIFT-1 or SWIFT-2) was also included as an additional covariate. A greater reduction in SNOT-22 score indicates a greater improvement in patient outcomes. CI, confidence interval; CRSwNP, chronic rhinosinusitis with nasal polyps; FEV_1_, forced expiratory volume in 1 s; ICS, inhaled corticosteroid; LS, least squares; SC, subcutaneous; SE, standard error; SNOT-22, Sino-Nasal Outcome Test-22.

**Figure 5:**
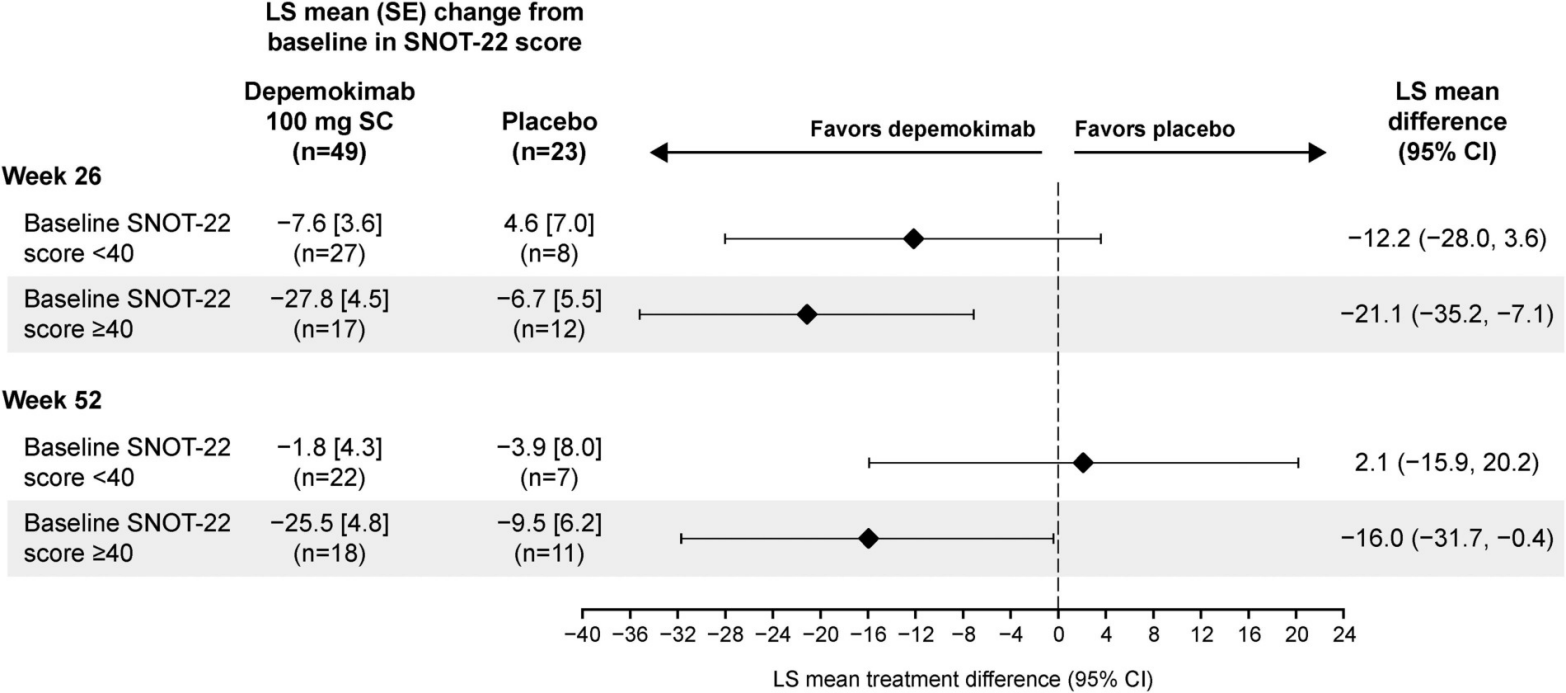
LS mean change from baseline in SNOT-22 score at weeks 26 and 52 in patients with type 2 asthma with current comorbid CRSwNP at baseline, by baseline SNOT-22 score (<40 and ≥40). *Only patients with current CRSwNP at baseline were included in the analysis. Analyses were performed using a repeated measures model with covariates of treatment group, baseline ICS dose (medium or high), exacerbation history (2, 3, 4+), geographical region, baseline SNOT-22 score, baseline pre-bronchodilator percent predicted FEV_1_, visit by baseline SNOT-22 total score and visit by treatment group (depemokimab or placebo). For the pooled analysis, study (SWIFT-1 or SWIFT-2) was also included as an additional covariate. A greater reduction in SNOT-22 score indicates a greater improvement in patient outcomes. CI, confidence interval; CRSwNP, chronic rhinosinusitis with nasal polyps; FEV_1_, forced expiratory volume in 1 s; ICS, inhaled corticosteroid; LS, least squares; SC, subcutaneous; SE, standard error; SNOT-22, Sino-Nasal Outcome Test-22.

The effect of depemokimab on SNOT-22 score was assessed in patients with current CRSwNP at baseline (*n* = 49 and *n* = 23 for depemokimab and placebo groups, respectively), with depemokimab demonstrating an improvement in LS mean SNOT-22 score at Week 26 compared with placebo in the asthma with CRSwNP subgroup [in patients with current CRSwNP at baseline; −15.1-point difference (95% CI: −25.0, −5.2)] and at Week 52 [−5.8-point difference (95% CI: −17.7, 6.1); Figure 4]. At both timepoints, the LS mean change from baseline in SNOT-22 score exceeded the MCID (8.9-point reduction) (20) with depemokimab, but did not exceed the MCID with placebo at either timepoint. Greater improvements with depemokimab vs. placebo in LS mean SNOT-22 score were seen in patients with a baseline SNOT-22 of ≥40 in the asthma with CRSwNP subgroup, compared with those with a baseline SNOT-22 of <40 (Week 26: −21.1 vs. −12.2-point difference; Week 52: −16.0 vs. 2.1-point difference, respectively; Figure 5), albeit with overlapping CIs. In addition, the odds of being a SNOT-22 responder [defined as an ≥8.9-point reduction (the established MCID for SNOT-22)] (20) was greater in patients receiving depemokimab vs. placebo in the asthma with CRSwNP subgroup (odds ratio ([95% CI]: 4.7 [1.3, 17.7] at Week 26 [61% vs. 30% responders]; odds ratio [95% CI]: 1.5 [0.4, 5.3] at Week 52 [43% vs. 35% responders]). These results should be interpreted with caution due to the small sample size and overlapping CIs.

## 4 Discussion

It is well-recognized that a significant proportion of patients with type 2 asthma have comorbid CRSwNP, which is associated with greater disease burden, making this an important subgroup to study. This pooled analysis of SWIFT-1/-2 data assessed the efficacy of depemokimab in patients with both conditions. The results demonstrated that twice-yearly depemokimab improved asthma outcomes vs. placebo in patients with both type 2 asthma characterized by blood eosinophils and CRSwNP (16). In particular, depemokimab vs. placebo resulted in a greater reduction in the rate of clinically significant asthma exacerbations in the asthma with CRSwNP subgroup compared with that observed in the asthma-only subgroup or in the overall pooled SWIFT-1/-2 population (16). In terms of asthma-related patient-reported outcomes, depemokimab also led to improvements in SGRQ and ACQ-5 scores vs. placebo in the asthma with CRSwNP subgroup, which were greater than those observed in the overall pooled SWIFT-1/-2 population (16). This enhanced efficacy of depemokimab in the asthma with CRSwNP subgroup may be due to the more pronounced type 2 inflammation typically seen in patients with comorbid CRSwNP, making them particularly responsive to targeted IL-5 inhibition.

Although depemokimab demonstrated consistent benefits across multiple clinical and patient-reported outcomes, no substantial improvement was observed in pre-bronchodilator FEV_1_ vs. placebo. This may in part reflect the variability and limitations of lung function as a primary marker of response in patients with type 2 asthma and comorbid CRSwNP. Although improvements from baseline in FEV_1_ were reported with depemokimab in the asthma with CRSwNP subgroup, a pronounced placebo effect was observed, as seen in the overall pooled SWIFT-1/-2 population, which reduced the treatment-placebo contrast and contributed to the lack of treatment difference. Such placebo effects in lung function measurements have been reported in other asthma studies and could stem from factors such as improved adherence to background therapy, increased clinical attention, or regression to the mean (21, 22). Another possible explanation may be the reversibility of airflow obstruction in this patient population, which may have reduced the sensitivity of FEV_1_ to detect a treatment effect. Overall, our findings suggest that while lung function remains a useful parameter, it may be less sensitive to treatment effects in populations with prominent type 2 inflammation, particularly when evaluating targeted biologics.

Improvements in upper airway symptoms, as indicated by improvements in SNOT-22, were observed with depemokimab vs. placebo in patients with current CRSwNP at baseline. However, the treatment effect on SNOT-22 was less pronounced at Week 52 and may be explained by a greater placebo response at this timepoint, which was not observed earlier in the study and may have diluted the treatment effect. This finding may reflect the small sample size and wide CIs observed, inherent variability in SNOT-22 scores, or other factors such as maintenance OCS use at baseline, a possible end-of-treatment effect, or increased patient tolerance to upper airway symptoms over time (23). Additionally, environmental and seasonal influences, such as allergen exposure or viral infections, may have contributed to symptom fluctuations but were not captured in the trial (24). These factors highlight the challenges of interpreting upper airway outcomes and underscore the importance of considering both clinical and contextual variables when assessing treatment impact in patients with type 2 asthma and comorbid CRSwNP.

When analyzed by baseline SNOT-22 category (<40 or ≥40), patients in the asthma with CRSwNP subgroup with a higher baseline score had numerically greater improvements in SNOT-22 at Week 52, despite limited patient numbers, suggesting a benefit in upper airway symptoms. This was consistent with findings from the Phase III ANCHOR-1/-2 trials, where greater improvements in SNOT-22 were seen in patients with baseline scores of ≥40 compared with the overall population (17), likely reflecting the higher potential for clinical improvement in those with the greatest disease burden.

Overall, these findings highlight the enhanced clinical benefit of twice-yearly depemokimab in patients with type 2 asthma and comorbid CRSwNP. Previous studies of other biologic therapies, including dupilumab (anti-IL-4/-13), omalizumab (anti-immunoglobulin E), benralizumab [anti-IL-5 receptor (R)], tezepelumab (anti-thymic stromal lymphopoietin), mepolizumab and reslizumab (both anti-IL-5), have demonstrated comparable or improved efficacy in patients with asthma with comorbid CRSwNP/nasal polyposis vs. those without or the general population (25–32). For example, mepolizumab has been shown to reduce exacerbation rates and decrease maintenance OCS use more effectively in patients with type 2 asthma and comorbid nasal polyps than in those without nasal polyps, compared with placebo (27, 33). Real-world evidence has also suggested that comorbid nasal polyposis or CRSwNP may serve as a predictor of 'super-responder' status in patients treated with mepolizumab (26, 34). In addition, although the anti-IL-5R benralizumab is not approved for CRSwNP, it has shown efficacy in both clinical trials and real-world studies in patients with type 2 asthma and comorbid CRSwNP or nasal polyposis, improving upper airway symptoms (e.g., SNOT-22) and multiple asthma outcomes, with evidence of greater responses observed in those with comorbid CRSwNP/nasal polyposis vs. those without or the general population (25, 28, 35). Taken together, findings from this pooled subgroup analysis and the wider literature indicate that targeting type 2 inflammatory pathways in patients with type 2 asthma and comorbid CRSwNP can potentially yield enhanced efficacy in both asthma-related and CRSwNP outcomes. It is important to note that in our analysis, patients with CRSwNP exhibited higher geometric mean blood eosinophil counts and a greater proportion received high-dose ICS at baseline than those who had asthma only, suggesting that CRSwNP may act as a marker of disease severity rather than being an independent driver of treatment response.

Strengths of this study include the robust, Phase III, randomized, controlled parent study population (SWIFT-1/-2), the pre-specified subgroup analysis and the inclusion of SNOT-22, which provides additional insights into upper airway outcomes. However, one limitation is that a small proportion of patients in the SWIFT-1/-2 population had comorbid CRSwNP (15%) and, with the 2:1 randomization, a small placebo comparator group and as such limited power. This percentage of patients with comorbid CRSwNP in the total SWIFT population is considerably lower than the proportions seen in the real-world SANI registry (∼40%) (5, 6), and is also generally comparable to or lower than the proportions reported at baseline in other randomized clinical trials of biologics for severe asthma (14%–23%) (36–41). This likely reflects the evolving patient population over time, whereby patients with type 2 asthma and comorbid CRSwNP, who experience the greatest disease burden are already on biologic therapy. As a result, the remaining pool of comorbid patients is likely smaller and potentially less severe, meaning fewer individuals are available for enrollment in clinical trials. Additional limitations were that patients in the SWIFT-1/-2 trials self-reported their past or current CRSwNP status, with no confirmatory endoscopy performed at study entry, and similar to most asthma trials, only one upper airway outcome was included (SNOT-22). The assessment of a range of upper airway symptoms in a larger population of patients with type 2 asthma and comorbid CRSwNP would be beneficial in future studies. In addition, the overlapping CIs observed between the CRSwNP and asthma-only subgroups suggest that CRSwNP modifies the treatment effect between subgroup specific estimates, rather than demonstrating a clear modification in treatment effect between the two subgroups. Nevertheless, despite these limitations, substantial improvements vs. placebo were observed on various clinical outcomes (both asthma- and CRSwNP-related), reinforcing the efficacy of depemokimab in this subpopulation.

In conclusion, results from this pooled subgroup analysis of the Phase III SWIFT-1/-2 data demonstrated that twice-yearly depemokimab reduced asthma exacerbation rates, SGRQ total scores, and ACQ-5 scores vs. placebo in patients with type 2 asthma and comorbid CRSwNP, exceeding those observed in the overall pooled SWIFT-1/2 population. While depemokimab provides clinical benefit across a broad asthma population, these findings suggest that patients with a higher type 2 inflammatory burden, such as those with comorbid CRSwNP, may experience enhanced treatment efficacy. This insight aids clinical decision-making, as the combination of comorbid type 2 asthma and CRSwNP is a clinical phenotype that can readily be identified and recognized when initiating patients with biologic therapy.

## Data Availability

The datasets presented in this article are not readily available. Please refer to GSK weblink to access GSK's data sharing policies and as applicable seek anonymized patient level data via the link https://www.gsk-studyregister.com/en/. Requests to access the datasets should be directed to https://www.gsk-studyregister.com/en/.
