# Supplementary material for: Depemokimab demonstrates efficacy in patients with type 2 asthma with comorbid CRSwNP: Phase III SWIFT-1/-2 analysis
Source: Front Allergy. 2026 Mar 6;7:1766647. doi: 10.3389/falgy.2026.1766647 (PMC13002596; doi:10.3389/falgy.2026.1766647)
Supplement: Supplementary file 1 [file Table1.docx]

Supplementary Material

# Plain language summary

# About 4 in 10 patients who have severe asthma also have a condition called chronic rhinosinusitis with nasal polyps. When these two diseases overlap, it is more likely that they have a type of inflammation which means that patients are more likely to experience a flare-up of symptoms and use oral steroids. As a result, these patients typically have a reduced quality of life. This analysis, which looked at data from two clinical trials, SWIFT-1 and SWIFT-2, investigated if patients with asthma and chronic rhinosinusitis with nasal polyps experienced benefits if they were treated with depemokimab two times per year. Patients who were at least 12 years old received either 100 mg of depemokimab by subcutaneous (under the skin) injection or placebo, every 26 weeks for 52 weeks. This study aimed to assess the rate of asthma flare-ups during the 52-week study period and quality of life by asking patients to complete health questionnaires. At the end of the study, a reduction in the rate of asthma flare-ups was shown with depemokimab compared with placebo. In addition, responses to questionnaires showed an improvement in quality of life and asthma control as little as 2–4 weeks after treatment and was sustained over the 52-week treatment period. The results of this analysis show that depemokimab administered twice per year is of clinical benefit to patients with asthma and chronic rhinosinusitis with nasal polyps, not only improving disease symptoms but also improving patient quality of life.

# Supplementary Data

**Figure E1. LS mean change from baseline in pre-bronchodilator FEV_1_ at Weeks 26 and 52 in patients with type 2 asthma with past or current comorbid CRSwNP at baseline**


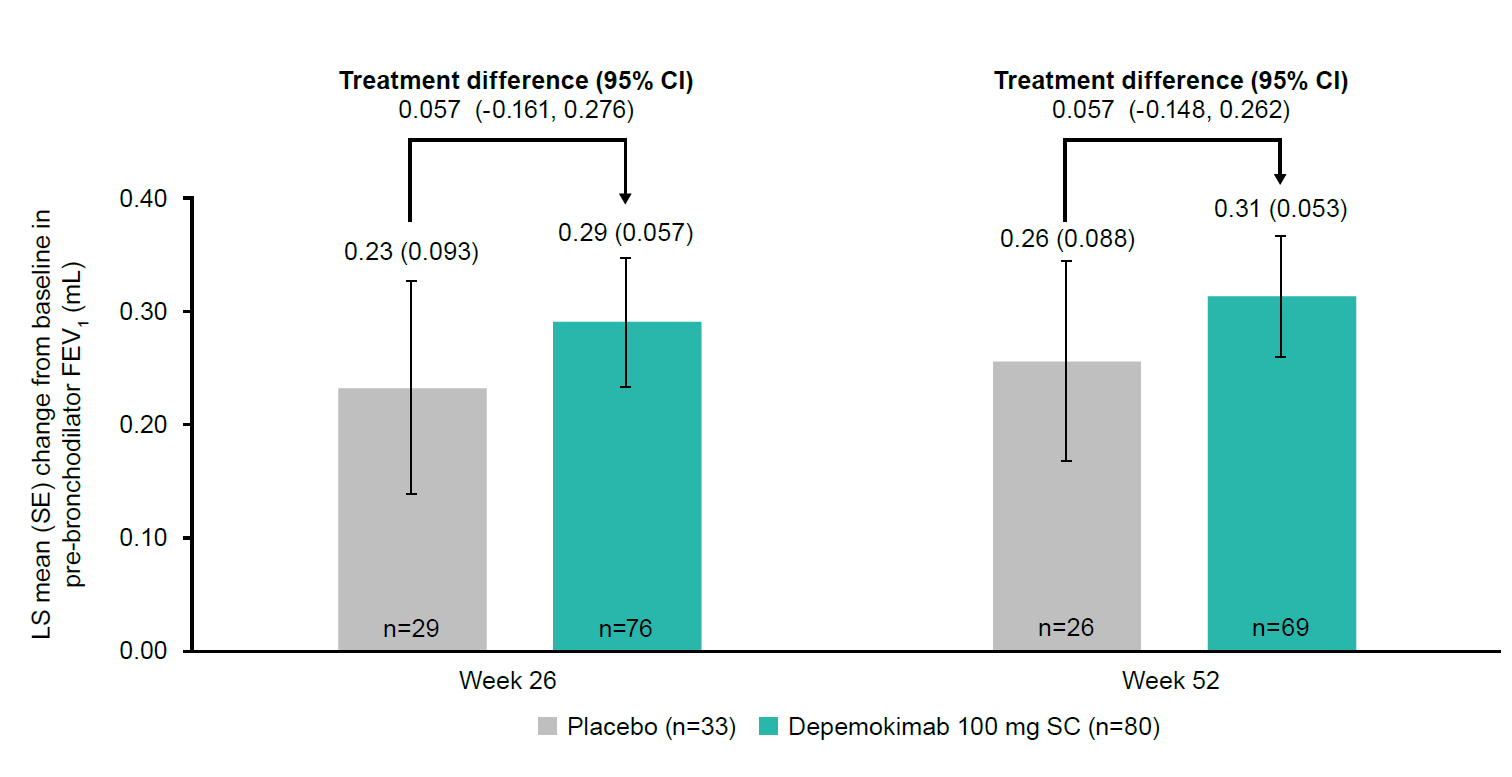


Analysis performed using a repeated measures model with covariates of treatment group, baseline ICS dose (medium or high), exacerbation history (2, 3, 4+), geographical region, baseline pre-bronchodilator FEV_1_, visit by baseline pre-bronchodilator FEV_1_ and visit by treatment group. For the pooled analysis, study (206713 or 213744) is also included as an additional covariate.

CI, confidence interval; CRSwNP, chronic rhinosinusitis with nasal polyps; FEV_1_, forced expiratory volume in 1 second; LS, least squares; SC, subcutaneous; SE, standard error.
